# Supplementary material for: A Theranostic Approach in SIRT: Value of Pre-Therapy Imaging in Treatment Planning
Source: J Clin Med. 2022 Dec 6;11(23):7245. doi: 10.3390/jcm11237245 (PMC9736029; doi:10.3390/jcm11237245)
Supplement: Supplementary file 1 [file jcm-11-07245-s001.zip › jcm-2036140-supplementary.pdf]

Table S1. Studies comparing the tumor and normal liver absorbed doses using MAA SPECT/CT to 90Y post treatment imaging using the Pearson coefficient for measuring the strength of the correlation.

Study	Type of microspheres	Post therapy imaging	Type of tumors	Nb of patients	Nb of tumors	TD correlation (Pearson coefficient)	NTLD correlation (Pearson coefficient)
Song et al. 2015 [30]	resin	^{90}Y PET/CT	HCC, CGC, mets	23	NA	0.64	0.71*
Gnesin et al. 2016 [31]	glass	^{90}Y PET/CT	HCC	7	11	0.56	0.99*
Gnesin et al. 2016 [31]	resin	^{90}Y PET/CT	HCC	20	30	0.74	0.93*
Haste et al. 2017 [35]+	glass	^{90}Y PET/CT	HCC	73	63	0.57	0.94
Kafrouni et al. 2019 [36]	glass	^{90}Y PET/CT	HCC	23	24	0.87	0.91
Richetta et al. 2019 [32]	resin	^{90}Y PET/CT	HCC	10	10	0.91	0.97
Jafargholi Rangraz et al. 2020 [33]	resin	^{90}Y PET/CT	HCC and mets	31	67	0.62	0.94
d'Abadie et al. 2021 [34]	resin	^{90}Y PET/CT	HCC and mets	66	171	0.65	0.97
Kokabi et al. 2022 [25]	resin	^{90}Y PET/CT	HCC	30	33	0.74	0.93*

HCC: hepatocellular carcinoma; CGC: cholangiocarcinoma; mets: metastases; NA: not available; Nb: Number; TD: Tumor absorbed dose; NTLD: non-tumoural liver absorbed dose, * NTLD refered to the non tumoural liver only targeted by SIRT + MAA SPECT performed without CT (no attenuation and scatter correction).

Table S2. Studies evaluating the accuracy of the prediction of tumor to normal liver uptake (TNR) with MAA SPECT/CT.

Studies	Type	Post treatment imaging	Number of patients	Number of tumors	Pearson coefficient
Debede et al. 2018 [37]	NA	^{90}Y SPECT/CT	12	12	0.9
Villalobos et al. 2021 [39]	HCC	^{90}Y SPECT/CT	190	NA	0.54
Son et al. 2021 [38]	HCC	^{90}Y PET/CT	34	45	0.79
Meine et al. 2021 [40]	HCC	^{90}Y PET/CT	22	31	0.71
Kokabi et al. 2022 [25]	HCC	^{90}Y PET/CT	30	33	0.53

NA: Not available; HCC: hepatocellular carcinoma
